# A 32-Channel Head Coil Array with Circularly Symmetric Geometry for Accelerated Human Brain Imaging

**DOI:** 10.1371/journal.pone.0149446

**Published:** 2016-02-24

**Authors:** Ying-Hua Chu, Yi-Cheng Hsu, Boris Keil, Wen-Jui Kuo, Fa-Hsuan Lin

**Affiliations:** 1 Institute of Biomedical Engineering, National Taiwan University, Taipei, Taiwan; 2 Athinoula A. Martinos Center for Biomedical Imaging, Massachusetts General Hospital, Charlestown, MA, United States of America; 3 Institute for Medical Physics and Radiation Safety, Dept. of Life Science Engineering, Mittelhessen University of Applied Sciences, Gießen, Germany; 4 Institute of Neuroscience, National Yang Ming University, Taipei, Taiwan; Le Fe Health Research Institute, SPAIN

## Abstract

The goal of this study is to optimize a 32-channel head coil array for accelerated 3T human brain proton MRI using either a Cartesian or a radial *k*-space trajectory. Coils had curved trapezoidal shapes and were arranged in a circular symmetry (CS) geometry. Coils were optimally overlapped to reduce mutual inductance. Low-noise pre-amplifiers were used to further decouple between coils. The SNR and noise amplification in accelerated imaging were compared to results from a head coil array with a soccer-ball (SB) geometry. The maximal SNR in the CS array was about 120% (1070 vs. 892) and 62% (303 vs. 488) of the SB array at the periphery and the center of the FOV on a transverse plane, respectively. In one-dimensional 4-fold acceleration, the CS array has higher averaged SNR than the SB array across the whole FOV. Compared to the SB array, the CS array has a smaller *g*-factor at head periphery in all accelerated acquisitions. Reconstructed images using a radial *k*-space trajectory show that the CS array has a smaller error than the SB array in 2- to 5-fold accelerations.

## Introduction

The quality of MRI critically depends on the radio-frequency (RF) receiver coils. While volume coils and surface coils provide a large field-of-view (FOV) and high signal-to-noise ratio (SNR) respectively, a phased array has been introduced to achieve both appealing features simultaneously by using carefully arranged surface coils and low-noise pre-amplifiers [1]. The high SNR offered by a coil array can also be traded-off for spatiotemporal resolution enhancement using the parallel MRI (pMRI) method [2], where different spatial sensitivity among channels of a coil array is used to estimate the skipped *k*-space data in acquisition by either an image domain [3] or a *k*-space algorithm [4, 5]. While there are versatile choices of reconstruction methods, the quality of the reconstructed MRI is still predominantly affected by the performance of the RF coil array [6].

One way to optimize the coil array in order to achieve high spatiotemporal resolution of pMRI is increasing the number of channels. Before reaching the theoretical limit [7, 8], increasing the number of channels typically improves spatial encoding by providing more versatile coil sensitivities. To this end, dense coil arrays for head imaging consisting of 16 [9–11], 32 [12, 13], 64 [14], and 96 elements [15] have been developed. There is also a cardiac array using up to 128 receiver channels [16]. The other approach to optimize the RF coil array design is to tailor its geometry to closely fit the imaging object such that the SNR can be maximized. This principle has been recently realized in, for example, a 32-channel lung array [17], an 8-channel wrist array [18], and 32-channel head coil arrays for pediatric imaging [19]. Independently, it has also been suggested that surface coils separated by a gap between them instead of overlapping neighboring ones can improve the quality of acquired data [9].

However, we consider that when the ultimate application of the coil array is pMRI, the coil geometry design should carefully consider the prescribed slice/volume orientation, the *k*-space trajectory, and phase/partition encoding directions in order to achieve the optimal performance. This is because the RF sensitivities will be an integral part of the spatial encoding in pMRI. A coil array providing the most disparate spatial information in RF coil sensitivities about the aliased image voxels in accelerated scans is expected to maximally suppress the aliasing artifacts due to sub-Nyquist sampling. One example demonstrating this rationale is reconstructing a two-dimensional image using a linear array with up to 64 elements using the single-echo acquisition [20].

Here we design a head coil array with elements distributed evenly over a two-dimensional phase-encoding plane in order to maximize the spatial encoding efficiency in pMRI acquisitions. While there has been 32-channel head array with coils arranged in a soccer-ball (SB) geometry [13] to allow for pMRI in three directions, the coil arrangement may be further optimized for pMRI in two directions. The coil geometry and arrangement of a circular symmetry (CS) array was designed to allow coil sensitivity evenly distributed over a two-dimensional phase-encoding plane in order to maximize the spatial encoding efficiency in pMRI acquisitions. We believe that such a design can improve the conditioning of the encoding matrix in pMRI when the skipped phase-encoding data are along the left-right and/or anterior-posterior direction. The benefit comes at the price of compromised sensitivity encoding along the head-foot direction and imaging depth. Such compromise brings us the flexibility in using coil sensitivity to better phase encode the spatial information along the left-right and anterior-posterior directions. Also, we minimize the size of the CS array in order to further improve the filling factor and thus to maximize the cortex SNR. We tested our array and compared its performance against the SB array using experimental measurements. We demonstrated this advantage using 1D and 2D Cartesian and radial *k*-space trajectory acquisitions. The CS array may also demonstrate similar advantages in highly accelerated 1D, such as inverse imaging [21], or spiral-trajectory acquisitions [22].

## Methods

### Coil design

Our circularly symmetric (CS) array consisted of 32 coils distributed over a head helmet with 21.5 cm and 18.5 cm clearance in the anterior-posterior and left-right directions, respectively. Each RF coil had a long trapezoidal shape and was curved to fit the head helmet from the vertex of the head toward the inferior part of the head. From the vertex view, RF coils were arranged in circular symmetry. The width of each RF coil was 3 cm at the inferior end and 1 cm at the vertex of the head. To allow presenting visual stimulation in future functional MRI experiments, the anterior coils were shorter than posterior coils in order to avoid obscuring the view of the subject. The length of the RF coil was either 14, 17, or 21 cm. The CS array had 10 short (14 cm) RF coils, 6 middle-length (17 cm) RF coils, and 16 long (21 cm) RF coils.

**Fig 1:**
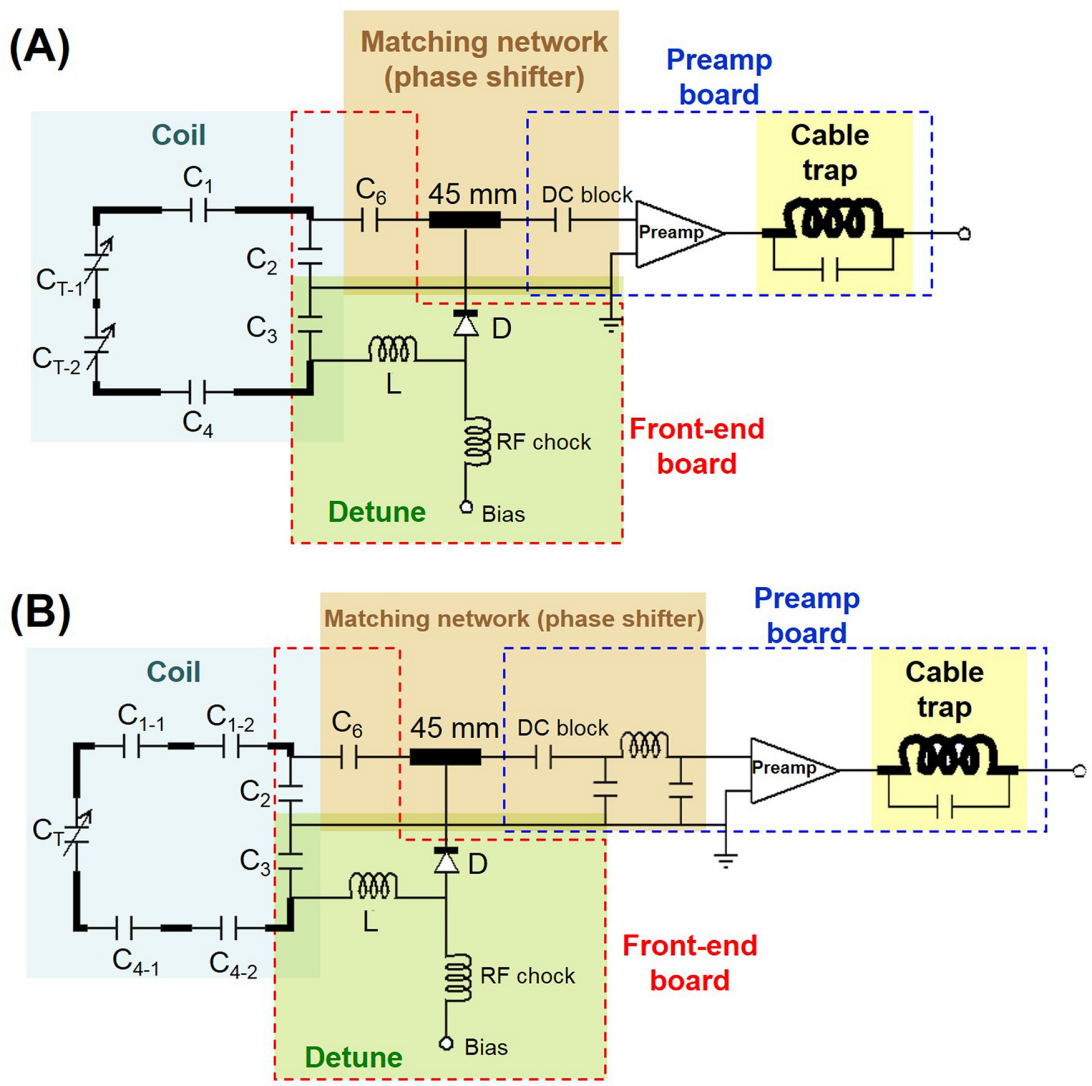
Schematic diagrams for a short (14 cm; A) and a middle-length or long (17 or 21 cm; B) RF coil.

To increase the quality factor (Q) and to decrease electromagnetic energy radiation [23], short RF coils were constructed with four distributed non-magnetic capacitors (Voltronics, Denville, NJ, USA) and two variable capacitors (Voltronics, Denville, NJ, USA) for resonance frequency tuning. We used the balanced circuit design in order to reduce the frequency shift under loading and to increase the Q [24]. Similarly, middle-length and long RF coils were constructed using a balanced circuit design with six non-magnetic capacitors and one variable capacitor. The coil used 16 AWG tinned-copper wire. Fig 1A shows the schematic diagram of a short RF coil, consisting of four capacitors (C_1_, C_2_, C_3_, C_4_) and two variable capacitor (C_T1, 2_). A 9×13 mm FR4 front-end circuit board consisting of a PIN diode D (M/A-COM Tech., MA, USA), an inductor L (Coilcraft, IL, USA), and a matching network with a capacitor (C_6_) and a 45 mm semi-rigid cable (UT-85C, microstock inc., PA, USA) was connected to C_2_ and C_3_. A DC block capacitor was connected between the 45-mm semi-rigid cable and a low-noise pre-amplifier. Pre-amplifier’s noise figure was less than 0.6 dB (typical value 0.5 dB) and the reflection coefficient was 0.950 +/- 0.03 with phase +150° +/- 3°. The output terminal of the low-noise pre-amplifier was connected to a cable trap to minimize the coupling between cables by suppressing the common mode current [25]. A cable trap with a high impedance was constructed by a hand-wound loop and a capacitor tuned at the Larmor frequency [24]. During RF transmission, the PIN diode D was forward biased and the parallel LC circuit (C_3_, L) was on resonance, consequently forming high impedance. Consequently, this parallel LC circuit blocked the current flow in the coil and detuned the coil during RF transmission. In the matching network, both the matching capacitor (C_6_) and the semi-rigid cable transformed the loaded coil impedance in order to achieve the optimal noise figure matching to the low-noise pre-amplifier. A middle-length or long RF coil used the detuning circuit, the matching network, the preamplifier and cable traps similar to those of a short RF coil (Fig 1B). Two minor differences were *i*) there were two more capacitors on the middle-length/long coil and *ii*) a π-network was added to transform the impedance of a middle-length or long RF coil to 50Ω.

**Fig 2:**
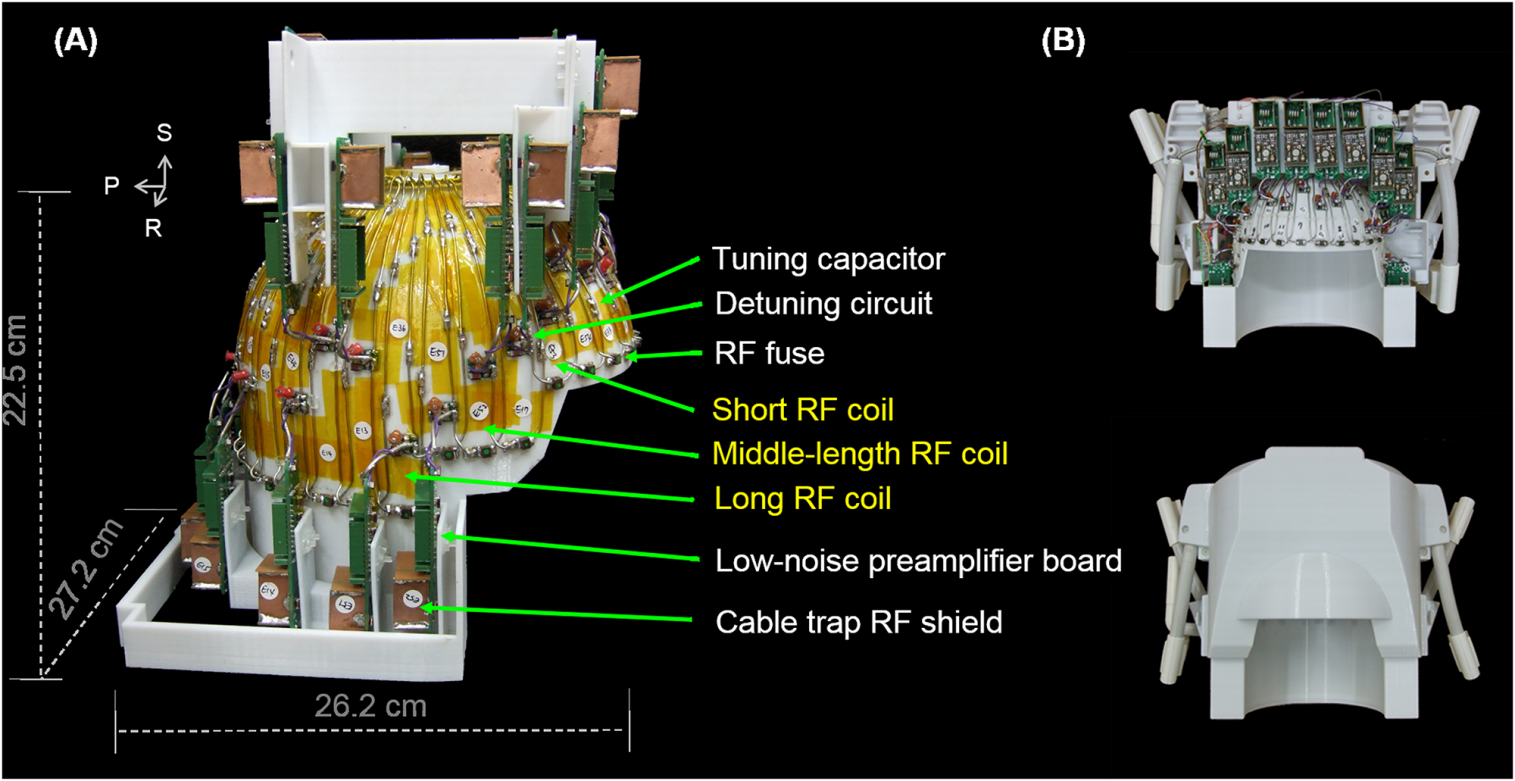
(A) Coil array dimension, circuits, and elements. S: superior; R: right; P: posterior. (B) The top view of the coil array together with the mechanical housing.

RF coils in an array need to be decoupled in order to obtain spatially independent information simultaneously. We chose to overlap neighboring RF coils to minimize the mutual inductance and thus to decouple the nearest neighboring RF coils [1]. There was significant coupling between next-nearest neighboring coils because coils were fairly close to each other. We used preamplifier decoupling to mitigate this problem [1]. Because the matching network also behaved as a phase shifter, which transformed the low input impedance of a low-noise pre-amplifier to high impedance, the induced current flowing on an RF coil due to coupling is thus minimized. In addition to the active detuning using a PIN diode, a fuse enduring up to 570 mA was serially integrated into the coil to ensure safety. All pre-amplifiers were arranged in parallel with ***B***_0_ in order to avoid the Hall effect [26]. Fig 2A shows the details of the array circuits.

The mechanical housing of the coil array was made by a 3D printer using polycarboneate (PC-ISO) plastic (FORTUS, Eden Prairie, MN, USA). The mechanical housing included a sliding machinery to allow the subject an easy access during scanning preparation. Fig 2B shows the top view of the array together with the mechanical housing.

### Bench measurements

We used an inductive probe formed by two decoupled loops (*S*_21_ < -60dB) to measure the resonance frequency of each RF coil. The Q value was measured either without loading or loaded with a spherical saline phantom. The *S*_21_ parameter was measured to quantify the mutual coupling between RF coils using a vector network analyzer (ZVL3, Rohde-Schwarz, Germany). The *S*_21_ parameter was also measured between two conditions when the coil was tuned and detuned in order to quantify the required detuning during RF transmission.

### Imaging experiments

All imaging experiments were done on a 3T MRI system (Tim Trio, Siemens Healthcare, Erlangen, Germany). Healthy human subjects participated this study. The study protocol was in line with the principles outlined in the Helsinki declaration. The study was approved by the ethics committee of National Taiwan University. A written informed consent was obtained from each subject prior to participation.

#### Detuning

To experimentally validate that coils were detuned properly, we measured B1^+^ map measured by a double-angle method [27] using EPI (flip angle = 60° and 120°, TR = 10 s, resolution = 4 mm isotropic). If coils were not detuned properly, the flip angle distribution would be inhomogeneous.

#### Noise covariance matrix, SNR, g-factor maps, and anatomical images

The coupling between channels of a coil array can be quantified by a noise covariance matrix, which was calculated from the acquired imaging data without any RF transmission using a 3D gradient echo sequence (FOV = 192 × 192 × 192 mm^3^, slice thickness = 1.50 mm, TR = 30 ms, TE = 3.5 ms, flip angle = 0°, BW = 260 Hz/pixel, image matrix = 128 × 128 pixels, 128 slices).

Given a noise covariance matrix **Ψ**, a SNR map was calculated [28]:

$$SNR_{\rho }=\sqrt{\frac{1}{{\left({\text{E}}_{full}^{\text{H}}\Psi^{-1}{\text{E}}_{full}\right)}_{\rho ,\rho }^{-1}}}\;,$$ (1)

where the matrix ***E***_*full*_ is the image encoding matrix corresponding to full gradient encoding satisfying the Nyquist sampling theorem based on the specified FOV and the nominal spatial resolution. The subscript *ρ* indicates the image voxel to be reconstructed, and the superscript *H* denotes conjugate transpose. The double subscript *ρ*, *ρ* denotes the diagonal element of a matrix corresponding to the image voxel *ρ*.

The amplification of reconstruction noise using accelerated pMRI data can be quantified by the *g*-factor [3]:

$$\begin{array}{c} \frac{SNR_{\rho ,\rho }^{accelerated}}{SNR_{\rho ,\rho }^{full}}=\frac{\sqrt{{\left({\text{E}}_{full}^{\text{H}}\Psi^{-1}{\text{E}}_{full}\right)}_{\rho ,\rho }^{-1}}}{\sqrt{{\left({\text{E}}_{acc}^{\text{H}}\Psi^{-1}{\text{E}}_{acc}\right)}_{\rho ,\rho }^{-1}}}=\frac{1}{g_{\rho }\sqrt{R}}\\ g_{\rho }=\frac{SNR_{\rho ,\rho }^{full}}{SNR_{\rho ,\rho }^{accelerated}\sqrt{R}} \end{array}\;,$$ (2)

where *R* denotes the acceleration rate, the ratio between the number of sampled data in full gradient encoding and that in the accelerated acquisition. ***E***_*acc*_ is the image encoding matrix in the accelerated acquisition. Here the encoding matrix consists of both the aliasing operation due to sub-sampling of the *k*-space data and the modulation of complex-valued coil-sensitivity. Note that encoding matrices were different when different *k*-space trajectories were used. The SNR and *g*-factor maps were calculated by discarding part of full gradient encoding (R = 1) data to simulate the undersampling in pMRI acquisitions.

#### Parallel MRI using a Cartesian *k*-space trajectory

**Fig 3:**
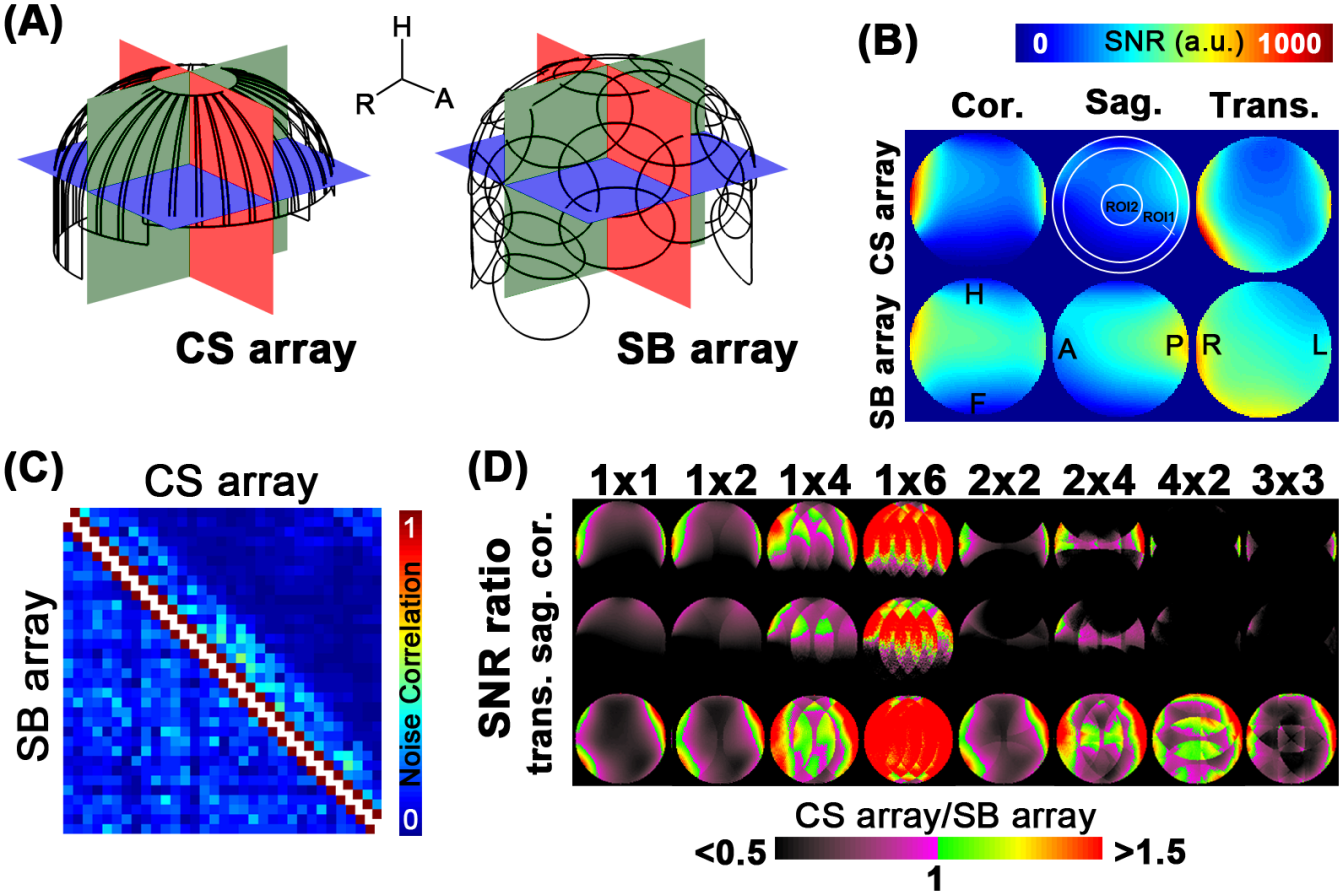
(A): coil geometries of both CS and SB arrays were arranged on the surface head helmet. The slice location of mid-sagittal, mid-coronal, and mid-transverse planes were shown. (B): The SNR profiles of the CS and SB arrays. The location of the ROI 1 and the ROI 2 were also shown in the sagittal slice image of the CS array. (C): The noise correlation matrix of both the CS array and the SB array. (D): SNR ratios comparison under 1D and 2D accelerated acquisitions using CS and SB arrays.

We first evaluated the performance of coil arrays in accelerated MRI using a Cartesian *k*-space trajectory with various 1D and 2D accelerations. Specifically, coil sensitivity maps were measured using a 3D GRE sequence (FOV = 192 × 192 × 192 mm^3^, slice thickness = 1.50 mm, TR = 30 ms, TE = 3.5 ms, flip angle = 15°, BW = 260 Hz/pixel, image matrix = 128 × 128 pixels, 128 slices) with a sphere saline phantom of 17 cm diameter. This phantom was placed at the center of the array. The evaluated region was set to a circle of 15.6 cm diameter within the phantom. Note that the size of the CS array was 18.5 cm in the left-right direction and SB array was 21 cm in the left-right direction. All reconstructions were performed on mid-sagittal, mid-coronal, and mid-transverse planes (see Fig 3A). Then, 2-, 4-, and 6-fold 1D accelerations and 2×2, 2×4, 4×2 and 3×3 2D accelerations were simulated. In this study, the *m*×*n*-fold acceleration represented accelerating the acquisition along the vertical and horizontal directions of shown images at *m* and *n* folds, respectively. Images were reconstructed with the SENSE approach [3]. Structural images demonstrating the use of the CS array were acquired using the MPRAGE pulse sequence with 2-fold acceleration and reconstructed by the GRAPPA algorithm (FOV = 256 6V256 mm^2^, slice thickness = 1.00 mm, TR = 2530.00 ms, TE = 3.03 ms, flip angle = 7°, BW = 130 Hz/pixel, image matrix = 256 256 pixels, 192 slices).

#### Parallel MRI using a radial *k*-space trajectory

To empirically evaluate the performance of the coil array in data acquisitions using a radial *k*-space trajectory, we simulated a radial *k*-space trajectory acquisition using data collected using a 3D GRE sequence. One- (180 radial lines), 2- (90 radial lines), 3- (60 radial lines), 4- (45 radial lines), and 5-fold (36 radial lines) acceleration acquisitions were simulated. Images were constructed by the *in vivo* sensitivity method [29] and the filtered back projection method, where the number of iteration steps was determined by controlling the residual error to be similar between the un-accelerated data acquired by the CS and SB arrays [30]. The performance of the reconstruction was quantified by calculating the error ratio at each image pixel: Error(**r**) = (R(**r**)-I(**r**))/I(**r**), where **r** denotes the index to an image pixel and R(**r**) denotes the reconstructed image pixel value. I(**r**) denotes the true image pixel value. Note that I(**r**) = 1 in the *in vivo* sensitivity method.

## Results

### Bench measurements

All coils were tuned to the resonance frequency 123.25 MHz. The ratio between unloaded and loaded Q for a long RF coil was approximately 268/83 = 3.23 ± 0.20, for a middle-length RF coil was approximately 212/81 = 2.62 ± 0.35, and for a short RF coil was approximately 176/78 = 2.26 ± 0.22. The *S*_12_ between two nearest-neighbor RF coils was lower than -12 dB under the loaded condition without preamplifier decoupling, which suggested good decoupling. The *S*_21_ difference between tuned and detuned coil was around 35 dB. This indicated that the RF coil was sufficiently detuned during RF transmission to avoid interference. The preamplifier decoupling reduction is approximate 23 dB.

### Imaging experiments

#### Coil detuning

B1^+^ map measured from the center slice of a saline phantom showed homogeneous flip angle distribution (59.97° ± 0.23°). Homogeneous flip angle distribution suggested that all coils were properly detuned and there was no significant interference from receiver coils to the transmit coil.

#### SNR maps, noise correlation matrix, and anatomical images

SNR maps were evaluated at three orthogonal planes as depicted by Fig 3A. Fig 3B shows the SNR maps for both CS array and SB arrays at three planes. We arbitrarily defined two regions-of-interest (ROI’s) to quantify SNR: ROI 1 represented a ring area around the periphery of the phantom with width of 1.2 cm. ROI 2 represented a circular area at the center of the phantom with radius of 2.4 cm. The SNR at the transverse plane for the CS array was 94% and 59% of the SB array in ROI 1 and ROI 2, respectively. The averages and standard deviations of SNR at ROI 1 for the CS and SB arrays were 473+/-182 and 501+/-137, respectively. The maximal SNR at ROI 1 for the CS and SB arrays were 1070 and 892, respectively. The averages and standard deviations of SNR at ROI 2 for the CS and SB arrays were 265+/-16 and 449+/-22, respectively. The maximal SNR at ROI 2 for the CS and SB arrays were 303 and 488, respectively. The SNRs of both arrays were comparable at approximately 3 cm from the surface of the CS array. Details for SNRs at the mid-coronal and mid-sagittal plane were reported in Table 1A.

**Table 1:** Ratios of average SNR (A) and average *g*-factor (B) at two regions-of-interest using CS or SB array and a Cartesian *k*-space trajectory with 1D and 2D accelerations. Please note that ROI 1 represented a ring area with the width of 1.2 cm at the periphery of the phantom, and ROI 2 represented a circular area with the radius of the ROI equal to 2.4 cm at the center of the phantom.

(A) Averaged SNR ratios (**CS / SB array**)									
	ROI	1	1×2	1×4	1×6	2×2	2×4	4×2	3×3
cor.	ROI 1	0.822	0.858	1.035	1.545	0.611	0.638	0.377	0.378
cor.	ROI 2	0.553	0.577	0.866	1.549	0.34	0.55	0.16	0.105
sag.	ROI 1	0.561	0.566	0.699	1.014	0.4	0.442	0.261	0.288
sag.	ROI 2	0.527	0.538	0.797	1.413	0.351	0.654	0.168	0.182
trans.	ROI 1	0.943	0.979	1.233	1.713	1.026	1.165	1.19	1.023
trans.	ROI 2	0.589	0.628	1.032	1.771	0.649	0.985	0.985	0.64
(B) Averaged *g*-factor									
	**CS array**		1×2	1×4	1×6	2×2	2×4	4×2	3×3
	cor.	ROI 1	1	1.4	2.6	2.6	4.5	5.1	4.9
	cor.	ROI 2	1	2.8	15.7	2.7	7	24	20.1
	sag.	ROI 1	1.1	1.7	3.1	2.3	4	7	5.9
	sag.	ROI 2	1.1	3.8	18.9	2.6	5.3	28	16.3
	trans.	ROI 1	1	1.3	2.4	1.1	1.6	1.6	1.6
	trans.	ROI 2	1	2.6	13.6	1.1	3.1	3.3	4.5
	**SB array**		1×2	1×4	1×6	2×2	2×4	4×2	3×3
	cor.	ROI 1	1.1	1.7	4.9	1.1	1.8	2	1.5
	cor.	ROI 2	1.1	4.3	45.6	1.2	4.4	7.4	3.7
	sag.	ROI 1	1.1	2	5.7	1.2	2.2	2.7	2
	sag.	ROI 2	1.1	5.8	50.6	1.3	6.1	9.4	5.4
	trans.	ROI 1	1.1	1.8	5.1	1.1	2	2.1	1.8
	trans.	ROI 2	1.1	4.7	41.7	1.2	5.2	5.7	4.8

Fig 3C shows the noise correlation matrix of both the CS array and the SB array. Quantitatively, the average and the maximum of off-diagonal entries for CS array was 0.09 and 0.50 respectively and for SB array was 0.14 and 0.37 respectively. Note that for the noise correlation matrix of the CS array, values corresponding to neighboring coils were shown at neighboring columns of the correlation matrix.

The SNR comparisons between two arrays with 1D and 2D accelerations using a Cartesian *k*-space trajectory were shown in Fig 3D. The SB array had higher SNR than the CS array for un-accelerated and 2-fold 1D accelerations. The CS array had slightly SNR advantage starting at 1D 4-fold acceleration. This advantage was only observed in the transverse plane, while there are significant SNR loss at the coronal and sagittal planes. The CS array provided nearly 72% and 78% SNR improvement than the SB array at ROI 1 and ROI 2 in 1D 6-fold acceleration at the mid-transverse plane, respectively. For 2D accelerations, the CS array outperformed the SB array in all acceleration rates at ROI 1 in the mid-transverse plane, while the SB array had a higher SNR than the CS array at all other combinations of ROIs and accelerations at the mid-sagittal and mid-coronal planes. Table 1A lists the ratio of SNR between CS and SB arrays at ROI 1 and ROI 2 for different 1D and 2D acceleration rates at three planes.

**Fig 4:**
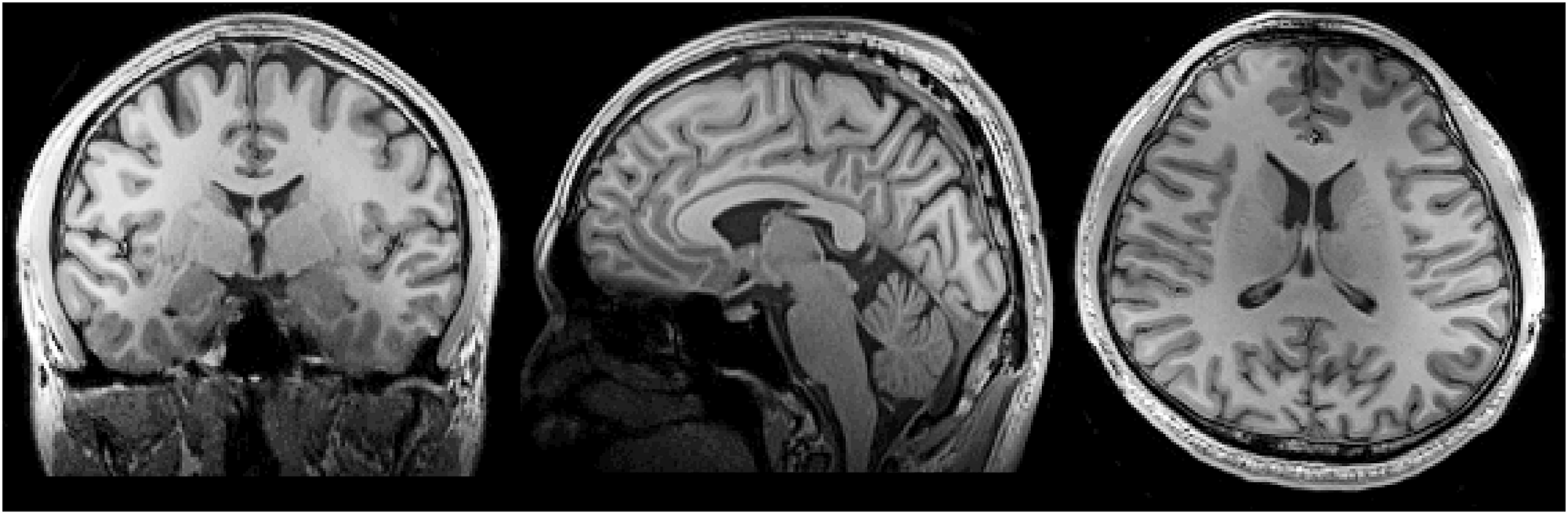
Structural images measured by the MPRAGE sequence at mid- sagittal, coronal, and transverse planes.

Fig 4 shows the anatomical images of a human head at the mid-sagittal, mid-coronal, and mid-transverse planes acquired with the CS array. High contrast between gray and white matters was observed in all images. While the SNR plot suggested that the CS array had lower SNR than the SB array at the center of the FOV, we still had good contrast and image quality at the thalamus, the medial aspect of the temporal lobes, and the cerebellum.

#### Parallel MRI using a Cartesian *k*-space trajectory

**Fig 5:**
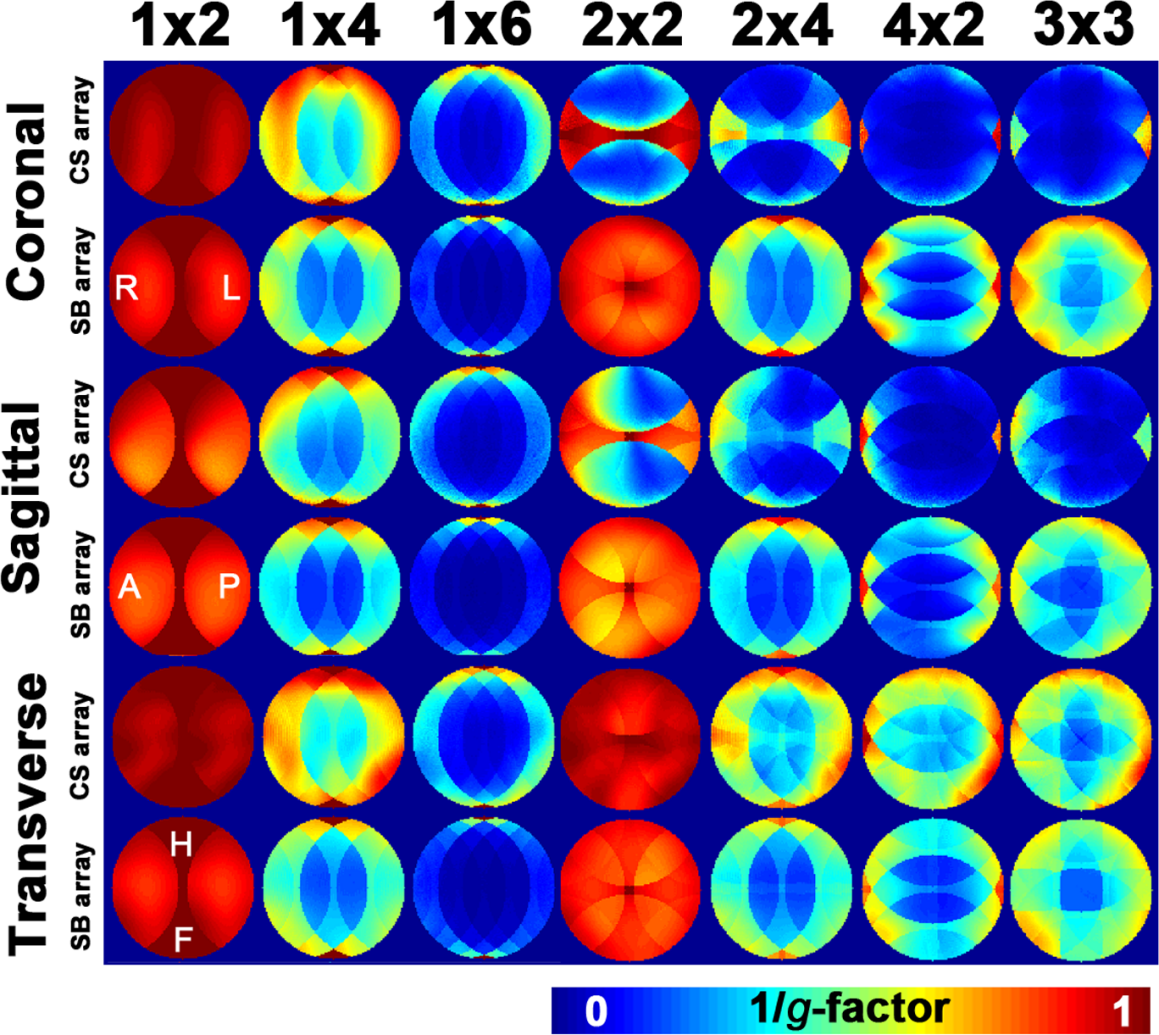
Spatial distributions of the noise amplification in 1D and 2D accelerated parallel MRI using a Cartesian *k*-space trajectory with CS and SB arrays. The noise amplification was quantified by the *g*-factor. The 1/ *g*-factor map was shown here.

Fig 5 shows spatial distributions of noise amplification (*g*-factor) in 1D and 2D accelerations. The most prominent advantage of the CS array in *g*-factor was observed at the mid-transverse plane images with 1D 4- and 6-fold accelerations. This advantage was also observed at the periphery of the FOV in 2D accelerations, where no acceleration was used along the head-foot direction. Importantly, the CS array cannot accelerate images along the head-foot direction, as clearly demonstrated by the high *g*-factor (small 1/*g* values) in 2D acceleration cases at mid-sagittal and mid-coronal planes (Fig 5). Details of the *g*-factors were listed in Table 1B.

**Fig 6:**
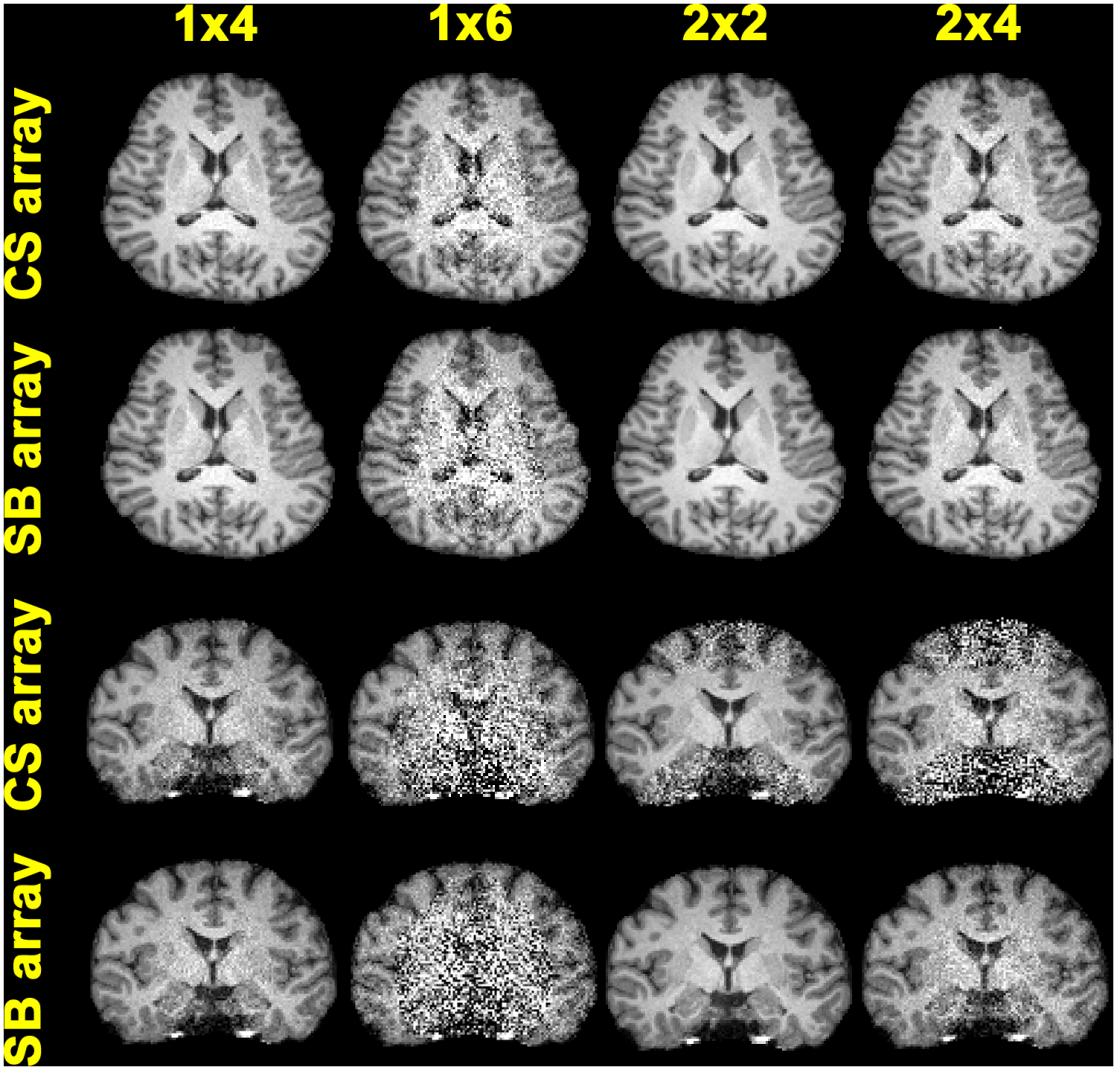
SENSE reconstructed images using 1D and 2D accelerated acquisitions with the CS and SB arrays.

Fig 6 shows SENSE reconstructed brain images with 1D and 2D accelerated acquisitions using the CS and SB arrays. At 1D 4-fold acceleration in the transverse plane, both CS and SB arrays generated similar reconstructed images. Clear difference between CS and SB arrays was observed in 1D 6-fold acceleration: the image from the CS array had much reduced noise than from the SB array. Images reconstructed from 2×2- to 2×4-fold 2D accelerations were found visually similar between the CS and SB arrays. The reconstructed images in coronal planes were also shown in Fig 6. The noise amplification pattern was similar to the *g*-factor map (Fig 5).

#### Parallel MRI using a radial *k*-space trajectory

**Fig 7:**
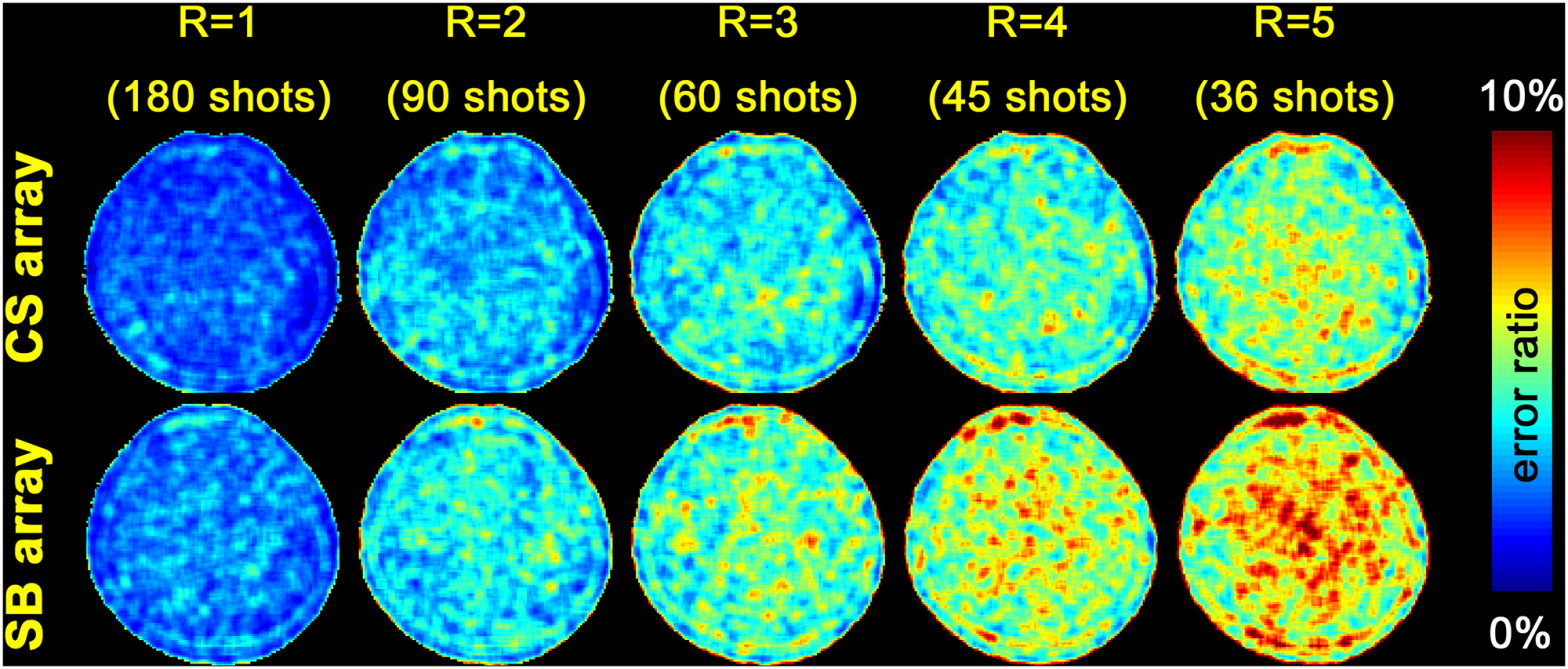
The spatial distribution of reconstruction error in 1, 2-, 3-, 4-, and 5-fold accelerated imaging using a radial *k*-space trajectory with CS and SB arrays.

We also evaluated the performance of CS and SB arrays in reconstructing accelerated acquisitions using a radial *k*-space trajectory. Fig 7 shows the spatial distribution of the reconstruction error. Compared to the SB array, the CS array shows smaller error.

## Discussion

Here we present a 32-channel head coil array with RF coils arranged in a circularly symmetric geometry. Our results show that, without any acceleration, the maximal SNR in the CS array was about 120% (1070 vs. 892) and 62% (303 vs. 488) of the SB array at the periphery and the center of the FOV, respectively. The most prominent advantage of the CS array was found in 1D 4-fold and higher accelerations, particularly at the periphery of the FOV (Fig 6). The reconstructions using measurements with a radial *k*-space trajectory in the transverse plane shows that the CS array has a smaller reconstruction error than the SB array at 2- to 5-fold accelerations (Fig 7).

The design of the CS array is similar to a previously reported 16-channel array [9]. Yet the differences are *i*) the number of channels in the coil array (32 channels in this study), *ii*) the method of decoupling the nearest neighboring coils (a gap *vs*. overlapping in this study), and *iii*) the demonstration of such an array in pMRI needing RF coil sensitivities evenly distributed over two dimension in order to maximally suppress the aliasing artifacts in image reconstruction of both 1D and 2D accelerated acquisitions. The advantage of this coil was particularly demonstrated in imaging using either a Cartesian or a radial *k*-space trajectory. Compared to the SB array, SNR advantage of the CS array was found at the periphery of the FOV in the transverse plane with SNR degradation (~40%) at the center of the FOV (Table 1A). The CS array also has advantages in SNR at the periphery of the FOV in the transverse plane (Fig 3D) and noise amplification (Fig 5) in accelerated parallel MRI using a Cartesian *k*-space trajectory. Anatomical images from the CS array were found less noisy than from the SB array in 1D accelerated acquisitions (Fig 6). Although the CS array has smaller noise amplification in 2×4 acceleration (Fig 5), the SB array has less noise at the central region of the brain in 2×4 accelerated image (Fig 6). This is likely related to the fact that the CS array had a lower SNR than the SB array in the center of the FOV in un-accelerated acquisition. Taken together, we found the CS array can be useful in pMRI suffering from high noise level in highly accelerated parallel acquisitions.

Compared to the SB coil array using the same number of RF coils, one obvious disadvantage of the CS array is its reduced sensitivity at the center of the FOV (Fig 3B). When the area covered by different arrays with the same number of coils is similar, it may be speculated that the combined sensitivity of each array is similar to each other regardless the shape of coil element. However, in practice, the resultant sensitivity depends on how coils are constructed, the intrinsic sensitivity depth of each individual RF coil, and how images are combined. These details critically determines the actual sensitivity of the measurements and the reconstructed images.

The other disadvantage of the CS array is that it cannot accelerate in the z-direction (head-foot direction), as clearly demonstrated in Fig 5. This is because the CS array does not have versatile coil sensitivity in the head-foot direction to help spatial encoding.

It should be noted that in our experimental comparison between the CS and SB arrays, their sizes were only close but not identical. The CS array had the 18.5 cm diameter clearance and the SB array was larger (21 cm diameter). This size difference may explain the difference in SNR reported in this study. However, it should be also noted that the SB array with slightly larger RF coils than those of the CS array has advantages of *i*) using larger elements with higher loaded/unloaded Q [13], *ii*) slightly farther separation among coils and thus making the off-diagonal entries of the noise covariance matrix smaller, *iii*) higher degree of freedom in placing pre-amplifier and thus the potential coupling between pre-amplifiers was reduced [19, 26]. These three advantages for the SB array were indeed the challenges of the CS array design and construction. While it could be argued that a CS array of the same size of the SB array would give fair comparison, we constructed the CS array such that it can tightly fit to the head in order to get the highest SNR at cortical regions for future applications, as demonstrated in the design of arrays tailored for pediatric imaging [19].

We expect that the CS array may also provide the same benefits in MRI using either spiral [31] or PROPELLER [32] trajectories, both of which have a point spread function distributed over the imaging plane. The similar advantage may be obtained in inverse imaging [21], which used 1D 64-fold acceleration, or single-echo acquisition using spiral-trajectory acquisition [22]. The other potential application of the CS array is O-space imaging, where a Z2 spatial encoding magnetic field is used for imaging [33]. At a transverse plane, the Z2 gradient cannot be used to localize magnetization at the same radius. Thus RF sensitivity distributed evenly azimuthally, such as the CS array, may help to accurately localize signals using pMRI methods.

## Conclusions

In summary, based on the rationale of designing a coil array with RF sensitivity tailored to efficiently mitigate the challenge of resolving spatial aliasing on a two dimensional plane in accelerated acquisitions, we designed, constructed, and tested the CS array with empirical data. This array can be a useful tool for experiments of high quality structural and functional imaging of human cortex when a Cartesian *k*-space trajectory or radial *k*-space trajectory is needed.
